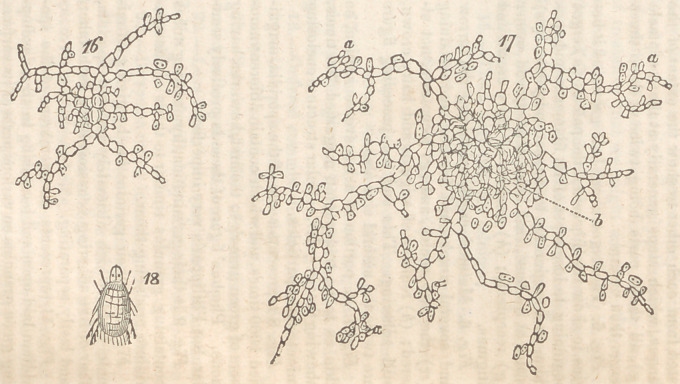# Foreign Summary

**Published:** 1839-08-24

**Authors:** 


### FOREIGN SUMMARY.

*Observations on the Anatomical and Physiological nature of the Ergot of Rye and some other Grasses.* By Edwin J. Quekett, Esq., F.L.S., &c., Lecturer on Botany at the London Hospital and Aldersgate School of Medicine.

[Continued fom page 532.]

Yet from this point, which is inseparable from the grain in the young state, it is most singular that in every kind of grass yet found ergotized, that the fungus should always burst through the tissue at this particular point, and at that particular time when the flower is about to expand. If it be a fungus solely, it ought certainly to burst forth as an ergot from the stem, or some other place on the several grasses, besides growing between and parting asunder two organs, which were as firmly united to each other in the young state, as the paleæ or glumes are to the same axis. Beside, the ergot, when matured like the ripe grain, slips out of the paleæ like a ripe filbert from its cupule, showing it has no organic connection at this period with the receptacle more than the grain had. Philippar's examination of the internal part seemed especially to strengthen his view of its being a fungus; for he describes the body of the ergot to be composed internally of branched short fibres, and globules of various sizes, round and oval, which he considered the means of its reproduction. My own observations on the structure of the ergot differ somewhat from this, by believing that the fibres described are the boundaries of irregular cells, distorted by the fungoid matter, and not fibres at all; and the globules are not reproductive bodies, but those of a fatty oil which is contained in the interior of the cells, as seen fig. 7, in a transverse section magnified 1000 times. To witness these facts, take an ergot, scrape away with a knife all its black coat, so as to remove all the particles that adhere to its surface, then to make some very thin transverse slices, and put them on a slip of glass under the microscope: and when water is added to them, it speedily becomes turbid or milky, from the quantity of particles that have escaped from the sections; these particles, however, are not heavier than the water, as those on the exterior of the ergot are, but are lighter, and collect on the surface, from whence they can be removed like cream from the surface of milk. When magnified, these particles are found to be of vastly many sizes, some as large as 1-1000th of an inch in diameter, others so small as to be barely visible when viewed to the extent that optical powers can assist us, and appear, when magnified, 1-1000th linear, very like the globules in human milk. When the water in which the slices have been placed is heated, these minute globules liquefy and run together, forming either very large globules or numerous irregular masses; their primary form, by this operation, being completely disturbed, which would not have been the case had they been "seminules," or reproductive agents, as Philippar imagined. To observe the structure of the ergot, make some thin slices, then boil them in ether, which dissolves the fatty matter, and makes their structure become visible, which is to all appearances irregularly cellular, and not fibrous.

Another argument against the ergot being a complete fungus is, that the particles which are its reproductive agents are most numerous when it is young, and it continues its growth after their production has ceased, which is contrary to the usual law amongst this class of vegetable productions; for their efforts to live are only to develope the means for propagation, dying, as it were, the instant this action has been accomplished.

Besides these, Vauquelin's chemical analysis proves its dissimilarity in composition with the FUNGACEÆ, and even with Sclerotium—a genus of that order to which the ergot was assigned by Fée and De Condolle—by containing very different constituents, which are the following:—

*Colouring matter*, soluble in alcohol.
*White oil*, very abundant, sweet.
*Violet matter*, soluble in water.
*Fixed phosphoric acid.*
*Azotized matter*, very abundant and alterable.
*Free ammonia*, at 100° Reaumur.

Thus far the arguments against the ergot being a species of fungus are taken from the body itself; but by experiments and minute examinations of the particles which separate from its surface, or are found in the viscid fluid which lodges externally, additional proofs can be obtained that corroborate the former views of its nature.

When these particles are placed under a microscope, and magnified about 1000 times (linear) their minute structure becomes then discernible, and their shape is seen to be oval or elliptical, and occasionally a little contracted about midway, and contain several green granules, whose number varies in different particles; most frequently there are one, two, or three well defined spots in their interior, and occasionally there are as many as ten or twelve; and there can be no doubt that these minute bodies are the reproductive agents of a particular fungus, to which particles the term *sporidia* is applied, to characterize them, because their structure is unlike seeds, notwithstanding their office is the same. Various conditions of these are seen at fig. 8.

FIGS. 6, 7, 8 and 9.

The size of these sporidia, upon an average, is about the 1-4000th of an inch in length, and 1-6000th of an inch in diameter, and the number on each ergot is uncertain; but as so many have been rubbed from one specimen as would fill a square inch of surface, it is probable, from the above measurement of their size, that about twenty millions may be calculated as an average number on a full-sized specimen; and as an example of the extreme minuteness of organic matter, some of these sporidia contain eight or ten granules, which are so small that it would require 200 millions of such to cover the same surface, their size not being more than 1-50,000th part of an inch.

If these sporidia be kept moistened with water on any suitable surface, or on a piece of glass, which is covered with a thin piece of talc, after a time it will be observed that these minute bodies commence germinating in various ways, and with me have continued to grow in this manner nearly three months.

The most common method is that of the sporidia emitting a tube or tubes from some uncertain point or points, (fig. 9,) but generally opposite the spot where a green granule is lodged in the interior. This tube increases to an indefinite length, and contains throughout its interior similar green granules, arranged at short but generally equal distances, about as far from each other as they are in the interior of the sporidia; and I believe that this tube ultimately separates into fragments, constituting as many new ones.

In many other instances, the sporidia, instead of producing a tube, give origin, opposite a green granule, to a minute bud; this little point increases, and ultimately separates from the parent as a perfect sporidium, and frequently before its separation shows an indication of producing a similar one from itself, (fig. 10.)

Another way of increase amongst these singular germs is, that of the membrane composing the parietes of the sporidium breaking down, forming a flat patch, (fig. 11,) which keeps extending in all directions, and developing upon itself green granules, such as are seen in the interior of the other sporidia. These granules seem important points, and appear to be analogous to the embryo of the seeds of more highly organized plants.

The last and most remarkable manner of germination is that of the sporidia, having a septum formed across their interior, by a green granule extending itself laterally, which divides them into two parts, each of which becomes again divided by a similar process, seen at figs. 12, 13,

14, 15. By a repetition of this method there at last is formed a moniliform filament, which, though simple in its origin, ultimately becomes branched, the branchlets most commonly radiating from a central collection of cellules. These filaments are the analogues of minute stems, and at a certain age give off, from innumerable points of their surface, little germs, which in a short time increase and become perfect sporidia, as seen figs. 16, 17 (*a a a*) which commence again in the several methods of germination just detailed. As the minute filaments belonging to one plant get what may be termed, ripe, the mass of cellules that have been developed about those first generated in the centre become to be considerably condensed and pressed together, as at fig. 17, (*b*,) so as to lose the distinct boundaries they originally possessed; and they begin to assume a brownish-yellow colour, and, in fact, look now exactly like a section of the body of the ergot itself.

Here, then, has been witnessed by daily examinations, the growth of these sporidia, which, being found on the ergot of every grass, are without doubt connected with the cause of its origin; these examinations show their various methods of germination, and their advancement to maturity and ultimate ripening, or producing the means of their reproduction; yet this minute plant does not measure more than 1-300th to 1-100th part of an inch in length or breadth.

The fact of having caused these minute plants to grow, independent or not connected with the body of the ergot, and without assuming any form in the least way similar to it, is the most convincing proof that the flocci, or arachnoid filaments, and the particles, before mentioned, occurring on the surface of the ergot, are no part of that body, but are the microscopic plants just described, which choose the grains of many grasses as the matrix of their developments, such plants belonging to the order of vegetables denominated Fungaceæ.

There are other proofs of the independent existence of the microscopic fungus, for it is found that it is not exclusively confined to the grain as a locality, but is observed to flourish on many other parts of the same grass, viz. in the interior and on the exterior of the anthers, on the paleæ, on the glumes, and on several parts of the rachis of the infected plant; but not occasioning there any exuberant growth of the part, for obvious reasons; because these parts have completed their development before the fungus makes its appearance; and their structure is not like that of the grain, which, at the period of the attack is exceedingly young, and just commencing to grow rapidly, and susceptible of impressions which can easily pervert its form and structure.

I conceive from the foregoing remarks that my examinations have proved that the ergot of the rye, as well as other grasses, is produced by a particular species of fungus, which developes itself upon or in the grain, whilst the latter is very young, causing its remarkable alteration from a healthy grain, in form, colour, chemical composition, and properties.

The method by which this singular production probably originates (for at present all respecting this part is uncertain) is, that the sporidia of this fungus are by some means introduced into the interior of the plant, and ultimately arrive at the grain, which they find the most suitable matrix for their development, or they are brought into contact with the young grain by some means (probably by the fluid) from without. In either case, when they come into contact with the grain, they lose no time in the work of reproduction, emitting their filaments through the tissue of the grain, and covering its body with multitudes of arachnoid filaments bearing sporidia, and apparently destroying its coats, as the matured ergot possesses no envelope.

Their presence communicates disease most frequently to the entire grain; sometimes, however, I have thought that the embryo only has been diseased, a part of the albumen remaining, along with the hairy tuft, on the apex of the ergot. This diseased action does not, I imagine, entirely deprive the grain of the power of growth, for it lives after the effects of the parasite have ceased: but it vitiates all its constituents, for neither starch nor gluten now exist, but instead, abundance of oil, which I suspect is produced by the grain, as none is seen from the microscopic plants whilst germinating in the way already described. As the ergot increases in size, it is made up partly of the diseased structure of the grain, and the fungic matter which has grown within it, which is like that observed when the parasitic plant grows unconnected with the grass, not being sporidia, but condensed cells such as compose the filaments, as at *b*, fig. 17.

To state my opinion, derived from experiments and examinations, which have been made and repeated again and again, in order to obviate every source of error arising from the manner in which they have been conducted, I would say, then, that I consider the body known as ergot to be a mass composed of the constituents of the diseased grain, mixed with fungic matter, occupying the place of the healthy ovary, of which can be observed some retained relics in its triangular shape, and the furrow on one of its sides, both conditions being those of the perfect grain also.

Since it has been, I trust, demonstrated that the ergot is no longer to be considered an independent fungus, it has become necessary to alter its previous botanical relations, by dismissing the former appellations, and giving a new one to the minute plant, which is the cause of this singular production.

From comparisons with the characters of the present little plant, and with those of British and foreign genera of Fungaceæ, it has been found so unlike any of them, as to deserve being made a new genus, to which I have given the title of *Ergotætia*;* and, after repeated examinations in the rye and other grasses, I have not hitherto found any material difference in the organization or characters of this parasite to warrant the making of those belonging to different grasses into different species, therefore I apply the specific term *abortans*† to the fungus found on the rye, and believe those on other grasses to be the same species.

This minute plant, from its structure and habit, will be classed in the suborder of Fungaceæ, *Coniomycetes* of Fries, and in the tribe of *Mucedines*.‡

Though many of these observations were primarily made with the elymus, because I had the plants in the growing state, yet the same experiments with the sporidia of the rye have been repeated, and with the same results, and the anatomy of the body of the ergot in both and in other grasses, seems to correspond in every respect.

This is a point which, as regards the goodness of the ergot of rye, is deserving mention in this place, from having found, in numerous instances, that the specimens have frequently been not much more than hollow cases, instead of being solid. On looking for the cause, it was found that these effects were produced by numbers of small acari, (fig. 18,) which devour the interior, thereby rendering such specimens nearly inert, and producing much powdery excrementitious matter about the ergot, similar to that observed with those species that dwell in cheese, or devour malted or other corn; therefore, the practice of keeping camphor, or some strongly smelling body with the ergot, is likely to be a preventive to the attacks of these tiny depredators.

17

16

18

† The term applies directly to the fungus destroying the germinating power of the grain, and indirectly to the medicinal properties of the ergot.

‡ In Berkley's arrangement of the British Fungi, *Ergotætia* will be placed in the suborder *Hyphomycetes*, and in the tribe *Sepidoniei*, which is composed of plants having filaments not sporidiferous; the sporidia being heaped together, and lying upon the matrix, which is nearly the case with the parasite of the ergot, whose filaments do not often bear sporidia, or if so, not one-hundredth time so frequent as the sporidia develope one from another, forming a mass which completely invests the body of the ergot.

*Explanation of the Figures.*

*Fig.* 1 represents the young grain of rye twice its natural size, (*a*) being the ovary crowned with hairs, (*b*); (*cc*) the feathery stigmas; (*d*) the place of the embryo; (*ee*) the two scales at the base of grain; (*ff*) lines representing the position of the paleæ, which are seen in their natural condition in fig. 3; (*g*) the pedicel or receptacle to which the grain is attached.

*Fig.* 2 shows the ripe grain of rye, twice magnified; (*a*) embryo; (*b*) crown of hairs; (*cc*) shrivelled stigmas; (*d*) albumen, composing body of the grain; (*g*) pedicel.

*Fig.* 3 shows ripe grain in its natural position between the paleæ (*ff*).

*Fig.* 4 is intended to give a representation of the commencement of the formation of the ergot; but an accurate idea cannot be well given, on account of the minuteness of the particles and filaments composing the fungus: (*a*) is the ovary of the grain overrun with the fungus, which completely hides it from the view; (*b*) shows the fungus has cemented the anthers and the stigmas together; (*ee*) the two scales at its base, separated from each other to show the extent of the fungus, which stops generally at the receptacle (*g*), all these parts being twice or three times larger than natural.

*Fig.* 5. The ergot about half grown as it begins to show itself between the paleæ; (*a*) ergot beginning to lose most of its filaments and sporidia, and beginning to appear purplish; (*ee*) scales at its base, that have been spread open; (*g*) receptacle; (*h*) remains of hairy crown and stigmas.

*Fig.* 6. Matured ergot, exhibiting the furrow and several cracks and fissures, and retaining (*ee*) the two scales, and (*g*) receptacle, not altered; (*h*) remains of stigmas and hairy crown, still adhering. This and the preceding figure are twice the natural size also.

*Fig.* 7. A portion of a transverse section, so thin as to be transparent, magnified 700 times, showing the irregular, cellular structure enclosing minute fatty particles.

*Fig.* 8 represents some of the sporidia, magnified 1000 times, and which contain different numbers of green granules; the first, however, having none. Phœbus' figure of these is precisely similar, but Philippar's very imperfect.

*Fig.* 9 is their germination, by emitting tubes containing granules similar to those of the sporidium producing them.

*Fig.* 10 is their germination by giving off minute buds, which ultimately become sporidia, four or five adhering occasionally to each other, and lastly separating.

*Fig.* 11 represents the membrane of the sporidium laid open and increasing in size, developing green granules on various parts of its surface.

*Fig.* 12 shows the manner a sporidium is divided by a septum or septa, by the green granules extending themselves laterally; different states being observed in the present figure.

*Figs.* 13, 14, 15. Different stages of the same process.

*Fig.* 16. The fungus assuming a radiating form, and beginning to develope sporidia upon its branches.

*Fig.* 17. The fungus arrived at maturity, its centre showing a structure analogous to that seen in fig. 7, and its several branches loaded with sporidia. Figs. from 8 to 17 magnified 1000 times.

*Fig.* 18. The acarus which lives on the interior of the ergot, being about one-fourth the size of the cheese-mite; magnified 80 times.

---

*Clinical Lecture on Hypertrophy of the Heart,—Dropsy,—Congestive Bronchitis,—Spurious Emphysema,—Cirrhosis,—Granular Kidney.* By Robert Carswell, M. D.—Gentlemen: I have to lay before you to-day the history of the case of John Davis, admitted with extensive disease of the heart, anasarca, and bronchitis. This patient had been under our care rather more than a fortnight, and, hopeless as his case was deemed by us, died suddenly, as not infrequently happens in such cases, without any very obvious aggravation of the symptoms.

The *post-mortem* examination of the body has furnished us with a number of very interesting examples of pathological alterations in several organs. The heart, the principal and primary seat of the disease, has afforded an example of hypertrophy of great extent, uncomplicated with valvular disease, endocarditis, or pericarditis. The lungs furnish another among the many examples which we have already met with of the congestive form of bronchitis; and of that form of pulmonary emphysema to which I have on several occasions directed your attention, as accompanying this complication, but which I have said is not the emphysema of Laennec and other authors. It ought not to be called emphysema, as it is not accompanied by dilatation of the air-cells, a circumstance which constitutes the essential physical character of vesicular emphysema. It consists simply in *inflation* of the lung, from the gradual accumulation and retention of the inspired air; in congestive and other forms of bronchitis, caused by the presence of the inspissated bronchial secretion; the swollen state of the bronchial mucous membrane; and, probably, by a modified condition of the vital contractility of the lung itself.

The liver and kidneys, also, in this patient, have presented us with specimens of morbid appearances with which few are familiar, and which, consequently, are either overlooked, or so imperfectly or inaccurately described, as not to be made available to the practical or scientific study of medicine. I shall read you the history of the case, and a description of the post-mortem appearances, before offering you any further remarks.

John Davis, aged thirty-three; admitted 15th May; naturally of slight conformation; sanguine temperament; a carpenter; married, and has a family of seven children; of very intemperate habits, being addicted to spirit-drinking for two or three years before his last illness. His relations on his father's side, and his sister, are stated to have died of consumption.

Healthy until the age of nineteen, when he had spitting of blood, brought on by carrying heavy pieces of timber; it occurred to a great extent, and was preceded by violent coughing. He was then confined to his bed for three months, and was subjected to active depletion. He afterwards recovered; a slight cough and dyspnœa remained; and the spitting of blood recurred, to a slight extent, on any strong exertion. Four or five years ago had spitting of blood, but not to the same degree as on the first attack, and was again bled, and completely recovered. Had gout on several occasions two or three years ago.

Last September twelvemonth he caught cold from exposure while heated, followed by cough and copious expectoration, sometimes streaked with blood; and had also tightness of the chest and dyspnœa. Was again bled, and employed other depletive measures for three months, which reduced him much in flesh and strength. Has had frequent recurrence of the cough and dyspnœa on exposure to cold; health best during the summer. At the end of September had violent headach during the day, the pain constantly shifting to all parts of the head and face. On recovering from this, at the end of three weeks, was again affected with great dyspnœa and slight cough, increased on the slightest exertion. Was under medical treatment, during which he used a bath of hot brine for his feet, to which he attributed the swelling of these and of the ankles, which appeared the following morning. The œdema gradually extended up the legs; these were punctured in various places, from which a quantity of fluid has been continually oozing, but without any reduction of the size of the limbs. The œdema is attended with great tenderness and burning heat of the skin, tingling, and a feeling of tension; it has gradually extended along the thighs and abdomen.

*Present symptoms.*—Skin hot and dry; feet cold by day, hot at night; ascites; œdema of feet, legs, and parietes of abdomen. The former feel very tense, and pit deeply on pressure; the skin is of a pale, waxy aspect; excoriation of the legs from the discharge from the punctures; countenance anxious and pale; dimness of sight; hoarseness from recent cold; breathing short, and affected by slight exertion; orthopnœa; slight cough, and expectoration of a muco-purulent matter.

*Physical signs.*—Impulse of heart too strong and too extended; bellows-sounds at base of heart, first slight, second louder; a trace of morbid sound at apex, with first sound.

Respiration anteriorly puerile, and slightly sonorous in upper part of right lung; slightly mucous and feeble inferiorly; large muco-crepitant rattle all over anterior part of left lung; posteriorly, respiration in right lung distinct near the vertebral column, about the middle of the back, in the rest of the lung very feeble, (almost wanting,) and slightly sonorous; and a little mucous inferiorly; left lung, muco-crepitant rattle to the bottom; percussion dull, over a large extent, in cardiac region transversely, over a less extent perpendicularly; clear in both lungs anteriorly, but clearer in the right lung than in the left; posteriorly duller in the lower third on both sides; clear in the upper two-thirds; dulness over a less extent than usual in the region of the liver; the left side of the chest appears smaller than the right; pulse ninety; tongue clean and moist; appetite good; diarrhœa with tenesmus immediately after taking food; urine scanty, and high-coloured and scalding; hæmorrhoids, and occasional discharge of blood.

The diagnosis in this case was not difficult; the physical signs of hypertrophy of the heart were conspicuous, and the morbid sounds heard in the region of this organ rendered it probable that it was complicated with valvular disease. The anasarca and ascites were the obvious consequences of the obstacle to the venous circulation, and partly of the state of the lungs, in which we had bronchitis and spurious emphysema, as dependent on the diseased state of the central organ of the circulation. The extent of the primary disease, and its grave complications, rendered the prognosis extremely unfavourable, and afforded a slight chance of success to any means of treatment that we might employ. The free state of the bowels, and the suffering he experienced from hæmorrhoids, prevented us from having recourse to drastic purgatives, and induced us to place our chief reliance on diuretics; he was, therefore, ordered the following diuretic medicines:—

15.—Tinct. digitalis, ten minims;
Tincture of squills, twenty minims;
Nitrous spirit of ether, one drachm;
Solution of iodide of potassium, twenty-five minims;
Peppermint-water, one ounce and a half; every six hours. Middle diet.

17.—Urine rather increased in quantity, and pale; specific gravity, 1010, acid reaction and albuminous.

18.—Ordered common diet and meat daily.

20.—Complains of great thirst. Soda water, an ounce and a half, occasionally.

21.—Solution of iodide of potassium to be increased to thirty-five minims.

22.—Less fever, but complains of flatulence. Half a pint of milk daily; the soda water to be omitted.

23.—Dyspnœa rather increased, and complains of palpitation on the least exertion.

24.—Better, but still very weak; pulse quick. A lotion of decoction of bark and tincture of opium for the hæmorrhoids.

25.—Better, but the cough is increased at night; complains of sickness and flatulence after food; bowels relaxed, and stools watery; urine less in quantity. Solution of iodide of potassium increased to forty-five minims, and to have

Extract of henbane, three grains;
Compound powder of ipecacuanha, five grains;
Calomel, two grains; every night.

27.—Great anxiety of countenance, and tremor of the upper extremities; extreme debility; the cough and dyspnœa increased without the power to expectorate; pulse weak and slow.

28.—Early in the morning he complained of faintness and vertigo, and immediately after died.

*Examination of the body twenty-six hours after death.*

*External appearance of the body.*—Considerable œdema of the feet, legs, and thighs, and also of the penis and scrotum. Abdomen large, globular, tympanitic, except posteriorly, where the sound on percussion is dull, accompanied with slight fluctuation.

*Chest.*—The chest was first examined, in order to ascertain more correctly the relative position of the contained organs, which would have been altered by previously opening the abdomen, and allowing the escape of the effused fluid, together with the removal of the viscera.

On removing the anterior walls of the chest, the right lung was found in a state of full inflation; the left was much less distended, did not project forward like the right, and adhered intimately to the mediastinum, costal pleura, from the summit of the lung, down to about the fifth or sixth rib, and also to the pericardium and a portion of the diaphragm, by means of old, pale, cellular adhesions, some portions of which were in a fibro-cartilaginous state, where they united the upper lobe of the lung with the costal pleura. That portion of the pleural cavity on this, the left side, where there were no adhesions, contained about a pint of clear serosity. The right pleural cavity, the pulmonary and costal pleura of which were perfectly free and healthy, contained fully a pint and a half of a similar fluid.

The right lung, after its removal from the chest, and when held up in the hand, retained the same bulk which it presented when the chest was laid open. It felt extremely light, when compared with its great bulk,—did not present the dark venous colour of congestion, even posteriorly; and along the whole of its anterior margin was very pale, being, in this portion of its extent, much less vascular than natural. This portion of it, also, but particularly inferiorly, felt, when pressed, quite cottony, and yielded but very little crepitus. The greater part of the lung communicated a similar sensation, but in a less degree. No part of it, even the posterior and depending portions, could be said to be congested; nor was there even the usual œdematous state of these latter parts. The anterior, paler, and most inflated portions, contained, indeed, less moisture than in the natural state. Nowhere was there any trace of emphysema; I mean to say, no trace of dilatation of the vesicular structure of the lung was to be detected. The left lung, separated from its adhesions and removed, did not appear more than half the size of the right. It did not communicate the same cottony feel when pressed, and was slightly congested posteriorly.

The inferior portion of the trachea, and the bronchial tubes of the right lung, contained a large quantity of muco-puriform fluid, and the mucous membrane was greatly congested, and of a livid red colour. The bronchi of the left lung contained, also, a quantity of secreted fluid, chiefly of inspissated mucus, but was not congested.

Two or three of the bronchial glands on the left side contained a large quantity of cretaceous matter.

The pericardium contained between four and five ounces of clear serosity. The heart, at least double the natural size, occupied, transversely, a proportionate extent of the left side of the chest. It presented, on its external surface, at the apex, an opaline spot, about an inch and a half in breadth, presenting a rather rough surface; and a spot of a similar nature at the base of the heart, occupying a portion of the right ventricle and auricle, but double the extent of the other, and presenting a rougher and more unequal surface. The enlargement of the heart depended chiefly on hypertrophy of the left ventricle. The walls of this cavity, very firm, and paler than natural, were an inch in thickness towards the base of the heart, and at least half an inch at the apex; the cavity of the ventricle being enlarged, but not in proportion to the hypertrophy of the walls. The auricle on this side, not more capacious than natural, was, in some points, double the natural thickness. The left ventricle was rather large, its walls slightly hypertrophied; the musculi pectinati of the right auricle were largely developed, as well as the columnæ carneæ of the right ventricle; and the septum ventriculi thickened in proportion to the walls of the left ventricle. All the valves might be said to be perfectly healthy. The only preternatural appearance observed was a slight enlargement and hardness of the corpus aurantii of one of the sigmoid valves of the aorta. The endocardium was generally of its natural colour and transparency, except in some points of the mitral valve, at its base.

The right ventricle and auricle contained a large, flattened, rather solid, coagulum, consisting more of fibrine than blood. It passed from the auricle into the ventricle, and from the latter into the pulmonary artery, its two great, and several of its subdivisions. It adhered, or, rather, was firmly entangled between the musculi pectinati, the chordæ tendineæ and smaller columnæ carneæ. In the left ventricle and auricle the coagulum was much smaller, and composed chiefly of dark blood, partly fluid.

*Abdomen.*—Intestines greatly distended with air. Between two and three pints of clear serosity in the depending part of the cavity. Stomach, duodenum, and jejunum, contained a considerable quantity of the mucous secretion mixed with bright, yellow-coloured bile; the cœcum and transverse arch of the colon, a large quantity of semi-liquid fæcal matter of the same colour.

The mucous membrane of the stomach presented in several points, and particularly where it formed rugæ, a punctiform redness. The mucous membrane of the duodenum was almost entirely of a deep and somewhat punctiform redness, and considerably thickened, and presented a round ulcer, about the size of a silver sixpenny-piece, midway between the pylorus and the orifice of the common bile-duct. The mucous membrane of the cœcum also presented a similar red colour, but much less in degree, and not general. In other portions of the intestine the mucous membrane did not present any preternatural appearance, except some congestion from depending position. The liver, the convex surface of which was on a level with the sixth rib, and adhered intimately to the diaphragm, presented externally the peculiar physical characters of cirrhosis. It was diminished in size, particularly the left lobe, and a number of flattened spheroidal bodies projected from its surface. These were of different sizes, varying from the size of a hemp-seed to that of a small split pea, and were much more numerous and conspicuous in some parts than in others. The liver felt harder than natural, and was of a dull, grayish-red colour. The whole of the interior of the organ presented the lobulated or tuberiform arrangement observed in the first stage of cirrhosis. The acini were grouped together in masses of various sizes, as seen on the exterior surface of the organ, and were separated from each other by a cellulo-fibrous tissue; and these, again, were divided into smaller ones by prolongations of the same tissue. The quantity of this tissue was greatest between the larger masses, and gradually diminished in their subdivisions amongst the acini. Its presence was, therefore, most conspicuous in the former situations, as a tissue foreign to the healthy structure of the liver, and distinguishable from it as well by its colour as its form and distribution. I shall take an early opportunity of pointing out to you the nature of this tissue, the manner in which it gives rise to the peculiar appearance of the liver named cirrhosis, the influence which it exercises on the portal circulation, and in the production of ascites. The spleen was considerably increased in bulk, probably twice the natural size. Both kidneys were small, slightly lobulated, very firm, and their proper covering opaque, thickened, and obscuring entirely the colour of the cortical substance, seen beneath in the natural state. When divided by a longitudinal section the cortical substance appeared rather pale, slightly granulated towards the circumference; and the tubular portion congested, accompanied by an indistinct appearance of its structure. On removing the serous covering of these organs, the granular appearance alluded to was much more marked, and was seen to be produced by closely-grouped, round bodies, of a whitish-gray colour, varying from the size of a mere point to that of a millet-seed. These were more closely set in some points than in others, and were rendered more apparent by the reddish-brown and somewhat vascular, unaffected, surrounding cortical substance. There were, likewise, a number of very small serous cysts, occupying, here and there, the external surface of both kidneys. The urinary bladder was healthy. The cellular tissue of the limbs presented, when cut into, the usual appearance found in mechanical œdema of this tissue. The brain was not examined.

With regard to these morbid alterations found after death, and the signs and symptoms by means of which we were enabled to detect their existence during life, it is necessary that I should make a few observations. The essential morbid condition of the heart was fully established, viz. that of great hypertrophy. The morbid sounds heard, and which were thought to depend, most probably, on valvular disease, were certainly produced by some other morbid condition of the heart, inasmuch as all the valves were found remarkably healthy. The rough patches observed on the surface of the heart were the only physical conditions which we met with likely to have occasioned them; and as they were situated in the regions of the heart where these sounds were heard, were, I am disposed to believe, the cause of them.

The morbid appearances found in the lungs, also corresponded with the physical signs which I have described. There was bronchitis, and there was spurious emphysema, or inflation of the right lung, marked by the clearer sound on percussion, and more fulness of the chest on this than on the opposite side. There was, however, hydro-thorax to a limited extent, and which, though indicated by dulness on percussion, was not named as depending on this state. The ascites was, of course, easily detected, although limited in extent; and, from the sound on percussion on the right side being clearer lower down than usual, although this might have depended on the inflated state of the lung, I thought it probable that there might be cirrhosis of the liver, a probability which was greatly increased by the intemperate habits of the patient. As the disease of the heart accounted fully for the ascites and anasarca, and as a coagulable state of the urine, with a low specific gravity, exists in dropsy of this nature, without disease of the kidneys, the pathological condition of these organs did not form an element in the diagnosis of this case. These organs, however, presented a marked example of the early stage of the granular kidney originally described by Dr. Bright.

The sudden cause of death in this case cannot excite your surprise when you reflect on the extent and quantity of the thoracic disease. The function of respiration ceased to be accomplished under a combination of causes. First, the accumulation of air, and the permanent inflation, particularly of the right lung, in the manner I have described; secondly, the obstacle caused by this to the circulation of the blood through the lungs, still more increased by, thirdly, the hydrothorax; and, lastly, the coagulation of the blood in the right side of the heart, which I am disposed to believe, from the appearance of the coagula, and the circumstances in which it occurred, took place previous to, and might have been the immediate cause of, death.

Before concluding, I may advert to the hæmoptysis which this patient experienced at an early period of his life, and which recurred on two or three occasions, at rather remote periods. The first and most severe attack followed violent muscular efforts in carrying heavy pieces of timber, and, I am disposed to believe, depended on bronchial, not pulmonary, hæmorrhage, or pulmonary apoplexy, as it is called. It is, also, equally probable that its occurrence was favoured, or that the predisposition existed, in consequence of previous disease of the heart. For hypertrophy of this organ is a frequent cause of hæmoptysis, particularly under the influence of the exciting causes which were in operation in this patient when it first occurred. Its subsequent occurrence obviously supervened on congestive bronchitis, of which he had several attacks, brought on by exposure to cold, and still more by the intemperate habits of the patient. That these, also, were complicated, on one occasion, with pleuritis, was shown by the old and dense cellular adhesions which existed so extensively on the left side of the chest. Lastly, I may remark that, in the history of the case of this patient, it is stated he lost several of his relations on his father's side, and his sister, from consumption. We did not find in the lungs any trace of tuberculous matter, or other alterations, dependent upon it, to afford us any evidence that he had had tubercular phthisis at an early period of life, at the age of 19, the period at which the hæmoptysis first took place, and to the presence of which the occurrence of hæmorrhage might have been ascribed. We found, however, evidence of tubercular disease having been present at a remote period in the bronchial glands. Several of these, situated along the course of the left bronchus, were completely filled with a hard, cretaceous-looking matter, such as is frequently met with in those who die of scrofulous disease of these and other organs of the body, and of tubercular phthisis. If the morbid condition which I have noticed was the consequence of scrofula, it is an example, among many which I have met with, of the curability of the disease, when confined to the lymphatic glands, established by the most unequivocal of all evidence,—that afforded by an examination of the affected parts after death.—*Lancet.*

---

*Death from pumping Air into the Eustachian Tube.*—An inquest was held in June last, by Mr. Wakley, coroner of Middlesex, on the body of an individual who died while undergoing an operation, which consisted in pumping air through the nostrils into the Eustachian tube. The following medical evidence relating to the subject, is found in the London Times of July 1st:

"Mr. James Reid deposed, that he found a thin layer of blood on the left side of the membrane of the brain, and globules of air under it, and in the small veins of the brain; that the left tympanum, or internal ear, had its lining membrane swollen, of red appearance, and there was a slight effusion of blood in it. From the known plethoric habit of the deceased, and from the fact of his having exerted himself at filling the air-pump before he was operated upon, he should say the cause of his death was apoplexy.

"Mr. Savage, lecturer on anatomy to Westminster Hospital, was next examined, and differed from the last witness, and stated that there was extravasated blood on both sides of the membrane, and that the tympanum of the right ear was affected as well as the left. He did not consider that deceased died of apoplexy, but that the injection of cold air through the Eustachian tubes was the primary cause of deceased's death. He knew that the instrument used by Dr. Turnbull would be affected by this opinion; but he did not think the operator in the case at all to blame, as he could not be aware of the nervous susceptibility of the patient.

"Mr. Liston, surgeon to University College Hospital, stated that he was present at the post mortem examination, at the request of the coroner, and the probability was that the deceased died in a continued fainting fit. He could not easily disconnect the forcible injection of cold air into the tympanum from the effect that followed it. In the region of the tympanum were a number of small nerves, connected with the most important one of the body, which, receiving an impression, would cause spasms, or other fatal affections of the heart. Nothing precisely satisfactory could be come to, on account of the decomposed state of the body."

A writer in the London Medical Gazette, and Lancet, recommends the observance of the following precautions, in the performance of the operation in question:

"Firstly. Under no circumstance ought the tube from the air-condenser to be accurately fitted to the catheter, one extremity of which is placed in the orifice of the Eustachian tube; but, as has been forcibly pointed out by the reviewer in the current number of the British and Foreign Medical Review, p. 95, the 'nozzle of the tube of the air-press should be held during the operation so loosely in the dilated end of the catheter, that there may be room for air to regurgitate.' By adopting this plan, although I have operated on my own ears many dozens of times, and upon patients many hundreds, I have never even produced emphysema, or any pain in the ear.

"Secondly. The condensed air must not be allowed to rush into the tympanum in the form of '*charges*,' but in a gentle and continued stream. Any one thinking of the peculiar and powerful effect produced in the ear, and over the whole of the head, during, and for some moments after, the distension of the cavity of the tympanum, by a forcible expiration with closed nostrils, can well imagine the result of a 'charge' from a powerful air compressor.

"Thirdly. No one ought to undertake the performance of the operation, who has not attained considerable dexterity and tact by the passage of instruments on the dead subject."

## Figures and Tables

**Figs. 6, 7, 8 and 9. f1:**
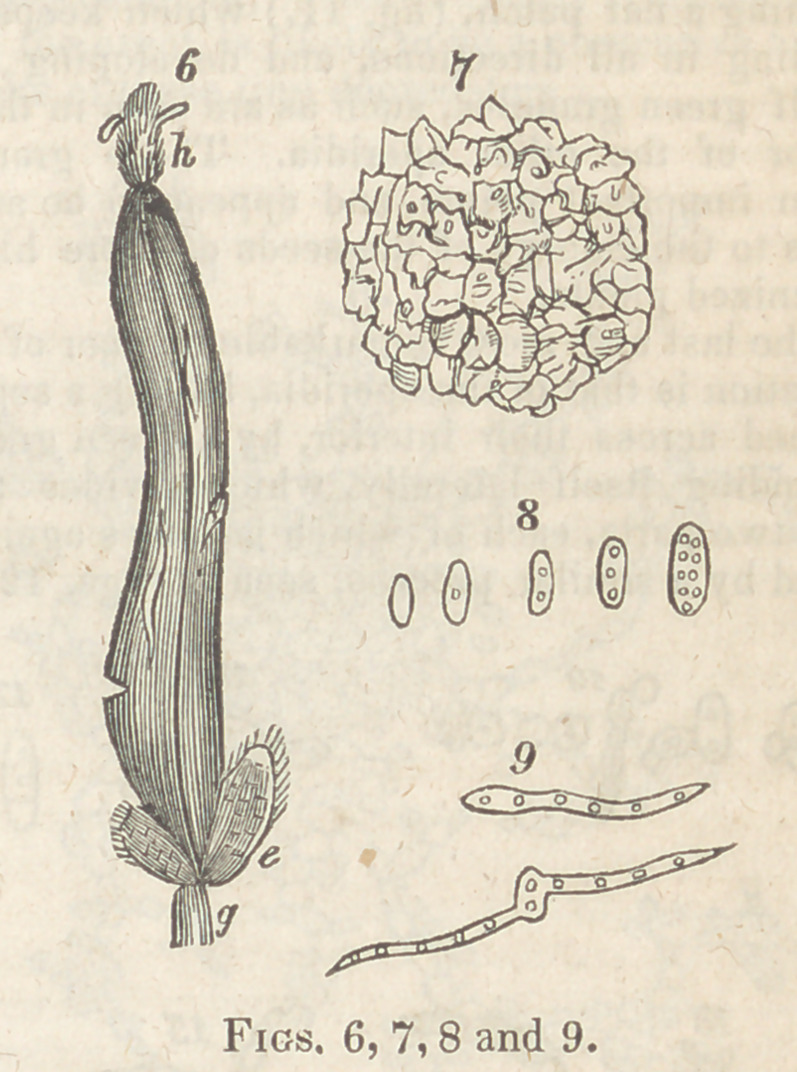


**Figure f2:**
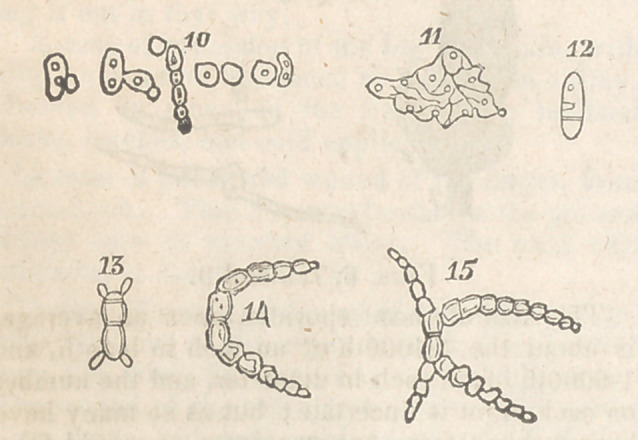


**Figure f3:**